# Thyroid function and non-alcoholic fatty liver disease in hyperthyroidism patients

**DOI:** 10.1186/s12902-021-00694-w

**Published:** 2021-02-18

**Authors:** Bairong Wang, Baomin Wang, Yumei Yang, Jing Xu, Mengyang Hong, Mingfeng Xia, Xiaomu Li, Xin Gao

**Affiliations:** 1 Present address: The Department of Endocrinology, Zhongshan hospital, Fudan University, NO. 180 Fenglin Road, Shanghai, 200032 China; 2 Department of Endocrinology and Metabolism, Jinjiang Municipal Hospital, Jinjiang, 362200 China

**Keywords:** Thyroid function, Non-alcoholic fatty liver disease, Hyperthyroidism

## Abstract

**Background:**

Although thyroid function has been demonstrated to be associated with non-alcoholic fatty liver disease (NAFLD) in different population, the prevalence and features of NAFLD in hyperthyroidism have not been reported. The present study aims to investigate the prevalence of NAFLD and association of thyroid function and NAFLD in hyperthyroidism patients.

**Methods:**

This cross-sectional study was performed in Zhongshan Hospital, Fudan University, China. A total 117 patients with hyperthyroidism were consecutively recruited from 2014 to 2015. Thyroid function and other clinical features were measured, liver fat content was measured by color Doppler ultrasonically, NAFLD was defined in patients with liver fat content more than 9.15%. Statistical analyses were performed with SPSS software package version 13.0.

**Results:**

The prevalence of NAFLD was 11.97% in hyperthyroidism. Patient with NAFLD had lower free triiodothyronine (FT3) and free thyroxine (FT4) levels than patients without NAFLD (*P* < 0.05). After adjusting for age, gender, metabolic parameters and inflammation factors, higher FT3 were associated with lower liver fat content (β = − 0.072, *P* = 0.009) and decreased odds ratio of NAFLD (OR = 0.267, 95%CI 0.087–0.817, *P* = 0.021).

**Conclusions:**

FT3 level was negatively associated with the liver fat content in this population. These results may provide new evidence in the role of thyroid hormone on the regulation of liver fat content and NAFLD.

## Background

Obesity and metabolic disease are becoming global issues, and the prevalence of Nonalcoholic fatty liver disease (NAFLD) is up to 20–30% [1–3]. NAFLD is a broad spectrum of diseases involves non-alcoholic simple fatty liver (NASFL), nonalcoholic steatohepatitis (NASH), liver cirrhosis, and hepatocellular carcinoma. NAFLD is becoming the main cause of cirrhosis, and NAFLD has been one of the major cause of the hepatocellular carcinoma [4]. Pathogenesis of NAFLD is not yet fully understood, it’s widely accepted that NAFLD is caused by the interaction of complex genetic background and environment factors [5], insulin resistance enhances the lipolysis of adipose tissue, impact a mass of free fatty acids (FFA) into the liver, leading to the deposition of FFA and triglycerides in the liver [6].

The thyroid hormone plays an important role in glucose metabolism, lipid metabolism, and insulin resistance [7, 8]. Emerging evidences have indicated the relationship between thyroid hormone concentration and NAFLD. Rochon et al. firstly reported the association between hypothyroidism and insulin resistance in 2003 [9]. Several other studies demonstrated that morbidity of NAFLD has an inverse association with thyroid hormone levels in the hypothyroid or euthyroid populations [10, 11]. Thyroid hormone analogues [11] and thyroid receptor beta antagonist [12] have been used to reduce liver fat content in animal models. Clinical studies also demonstrated that thyroid hormone analogues may improve NAFLD [13, 14]. However, there are no studies on the prevalence of NAFLD in hyperthyroid patients, and little is known about the association of thyroid hormone and liver fat content under hyperthyroidism condition. It should considered that the insulin resistance and oxidant stress [15] have been reported in patients with hyperthyroidism, both of which were related to the pathology of NAFLD. Therefore, it is necessary to explore liver fat content, NAFLD and possible mechanism in patients with hyperthyroidism.

The aim of the present study is to determine the association between thyroid hormone levels, and liver fat content in patients with hyperthyroidism and investigate the differences of clinical characteristics, including thyroid hormone levels, between patients with or without NAFLD. This study were conducted in 117 patients with hyperthyroidism, and liver fat content was measured by ultrasonography with our previous established methods [16].

## Methods

### Study population

The present study consecutively enrolled 117 patients with new-onset or recurrent hyperthyroidism from outpatient clinic in Department of Endocrinology, Zhongshan Hospital, between 2014 to 2015. The sample size was based on previous similar studies [17–19]. The Diagnose criteria of hyperthyroidism includes:1) hyper-metabolic syndrome include nervousness, irritability, increased perspiration, heart racing, hand tremors, anxiety, difficulty sleeping, thinning of the skin, fine brittle hair, muscular weakness, and other typical symptoms; 2) Laboratory tests show a low thyroid stimulating hormone (TSH), raised triiodothyronine (T3) or thyroxine (T4), and positive anti-TSH-receptor antibodies. 3) defuse increased radio-iodine uptake by the thyroid.

Exclusion criteria: 1) Patients with the history of taking medication that may affect thyroid function; 2) Hyperthyroidism crisis; 3) Hyperthyroid heart disease; 4) History of hypothalamus or pituitary disease; 5) Long-term alcohol consumption; 6) History of viral hepatitis, autoimmune hepatitis, Wilson’s disease; 7) Total parenteral nutrition, inflammatory bowel disease,or Cushing’s syndrome; 8) taking tamoxifen, amiodarone, sodium valproate, methotrexate, or glucocorticoids. Written, informed consent was obtained from all of the participants, and the study was approved by the ethics committee of Zhongshan Hospital, Fudan University, China.

#### Clinical and biochemical measurements

The clinical data including age, gender, history of drinking and alcohol intake were obtained from the clinical documents of endocrinology clinic. According to the routine protocol, the information of complete physical examination including height, weight, waist circumference (Wc), and blood pressure were recorded after overnight fasting for 12 h, and body mass index (BMI) was calculated by body weight (kg)/height squared (m^2^). The general laboratory tests (Japan Hitachi 7600 biochemical analyzer) included the levels of fasting blood glucose (FBG), total cholesterol (TC), triglyceride (TG), high-density lipoprotein cholesterol (HDL-C), low-density lipoprotein cholesterol (LDL-C), alanine aminotransferase (ALT), aspartate aminotransferase (AST), uric acid (UA). Free triiodothyronine (FT3), free thyroxine (FT4), TSH, thyrotrophin receptor antibody (TRAb), fasting plasma insulin (f-INS), and hyper-sensitive C-reaction protein (HCRP) were also measured (electrochemiluminescence, Roche Diagnostics, Germany). Insulin resistance index (HOMA-IR) were calculated by FBG × fasting insulin / 22.5. All subjects received a ultrasonic examination performed with a GE Vivid7 ultrasound machine as described previously [16]. Briefly, in the sagittal liver/right kidney view of ultrasound images, a region of interest (ROI) in the liver parenchyma and the kidney cortex was selected. In the right liver intercostals view of ultrasound images, a ROI in the liver far-field region was selected. And then all images were analyzed-of by using NIHimage software to estimate the gray scale mean value of the pixels within the two ROIs and calculated the hepatic/renal echo-intensity ratio and hepatic echo-intensity attenuation rate. A 3D abdominal phantom Model was introduced to standardize the measured values of US H/R ratio and hepatic echo- intensity attenuation rate, finally we can compute the liver fat content as the following formula: Liver fat content (%) = 62.592 × US hepatic/renal ratio + 168.076 × US hepatic attenuation rate − 27.863. NAFLDwas defined in patients with liver fat content more than 9.15% according to our previous research [16]. .

### Statistical analyses

Normal distribution variables were expressed as the mean ± standard deviation; Comparisons of normal distribution variables between the two groups were performed with the independent sample T test; one-way ANOVA test was used in comparison among the three groups. Non-normally distributed variables were expressed as the median (inter-quartile range), comparisons of non-normally distributed variables between the two groups were performed with the Mann-Whiteny test, while Kruskal-Wallis test was used in comparison of the three groups. The chi-squared test was used to compare the rates. The correlation between liver fat content and thyroid function and other clinical indexes was analyzed by Pearson correlation analysis. Multiple linear regression analysis was used to detect the independent correlation between thyroid function and liver fat content; statistical analyses were performed with SPSS software package version 13.0.

## Results

### General characteristics of the study population

The general clinical information all patients was showed in Table 1.The present study included 117 patients (34 males, 83 females), including 6 patients were subclinical hyperthyroidism. The average age was 45.35 ± 12.78 years, BMI was 22.55 ± 3.38 kg/m^2^. The prevalence of NAFLD was 11.97% in this hyperthyroidism population. Patients with NAFLD had lower FT3, FT4 levels (*p* < 0.05) and higher BMI, Wc, TG, TC, LDL levels (*p* < 0.05, respectively) than patients without NAFLD. There were no significant differences in age, gender, blood pressure levels, TSH, ALT, AST, UA, FBG, f-INS, IL-6, HCRP and HOMA-IR between the two groups (*p* > 0.05).

**Table 1:** Clinical characteristics of 117 cases with hyperthyroidism

Characteristics	Total (*n* = 117)	Without NAFLD (*n* = 103)	With NAFLD (*n* = 14)	*P*-value
Age (years)	45.35 ± 12.78	44.72 ± 13.04	50.00 ± 9.88	0.148
Gender(M/F)	34/83	29/74	5/9	0.559
BMI (kg/m^2^)	22.55 ± 3.38	22.19 ± 3.31	25.23 ± 2.72	0.001
Waist circumference (cm)	80.82 ± 9.52	79.93 ± 9.19	90.29 ± 8.06	0.005
SBP (mmHg)	122.52 ± 13.79	121.82 ± 13.77	127.71 ± 13.33	0.115
DBP (mmHg)	74.32 ± 9.35	73.73 ± 9.34	78.71 ± 8.50	0.115
FBG (mmol/L)	5.09 ± 1.08	5.04 ± 0.91	5.48 ± 1.92	0.159
f-INS (mU/L)	8.70(6.50,10.60)	8.70(6.20,10.55)	8.90 (7.35,11.63)	0.690
TG (mmol/L)	0.99 ± 0.49	0.94 ± 0.43	1.36 ± 0.71	0.048
TC (mmol/L)	3.45 ± 0.81	3.37 ± 0.76	4.00 ± 0.95	0.006
LDL (mmol/L)	1.79 ± 0.66	1.72 ± 0.62	2.26 ± 0.76	0.004
IL6(pg/ml)	2.30 (2.00,3.00)	2.30 (2.00,3.00)	2.10 (2.00,3.00)	0.564
HCRP (mg/L)	0.80 (0.40,1.98)	0.80 (0.40,2.05)	0.90 (0.70,1.70)	0.594
UA (umol/L)	292.89 ± 69.51	293.14 ± 66.50	291.07 ± 91.53	0.917
ALT(U/L)	36.80 ± 33.06	38.32 ± 34.45	25.64 ± 16.96	0.179
AST(U/L)	27.15 ± 16.61	27.86 ± 17.10	21.86 ± 11.50	0.206
FT3(pmol/L)	20.42 ± 12.73	21.69 ± 12.57	11.09 ± 10.01	0.002
FT4(pmol/L)	54.15 ± 29.87	57.11 ± 29.58	32.41 ± 22.73	0.002
TSH (uIU/ml)	0.005 (0.005, 0.010)	0.005 (0.005, 0.010)	0.005 (0.005, 0.508)	0.824
HOMA-IR	1.90 (1.38,2.54)	1.86 (1.37,2.52)	2.26 (1.56,3.22)	0.956

*BMI* Body-mass-index, *SBP* Systolic Blood Pressure, *DBP* Diastolic blood pressure, *FBG* Fasting blood glucose, *f-INS* Fasting insulin, *TG* Triglyceride, *TC* Serum total cholesterol, *LDL* Low density lipoprotein, *IL-6* Interleukin-6, *HCRBP* Hypersensitive C-reactive protein, *UA* Uric acid, *ALT* Alanine aminotransferase, *AST* Aspartate aminotransferase, *FT3* Free triiodothyronine, *FT4* Free thyroxine, *TSH* Thyroid-stimulating hormone, *HOMA-IR* Homeostasis model assessment for insulin resistance

**Fig. 1:**
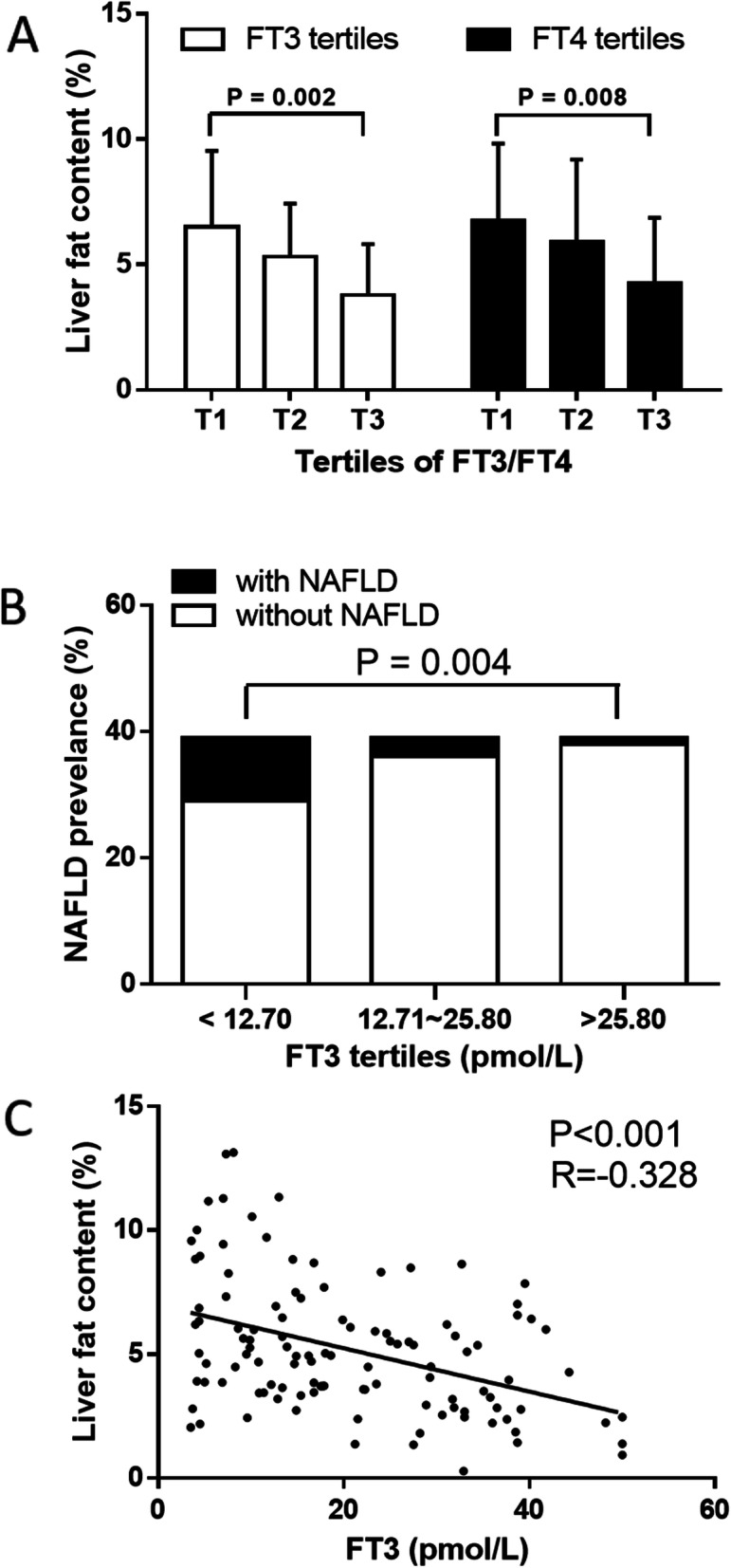
Correlation of liver fat content and prevalence of NAFLD with thyroid hormoe levels in hyperthyroidism patients. **a** The FT3/FT4 levels in respective tertiles was plotted against liver fat content, the liver fat content showed a decreased trend with increasing of FT3/FT4 levels; **b** The FT3 levels in respective tertiles was plotted against prevalence of NAFLD, the prevalence of NAFLD showed a decreased trend with increasing of FT3 levels; **c** The FT3 concentration was plotted against liver fat content, and a negative significant correlation was found between FT3 and liver fat content

All subjects were further divided into three groups according to tertiles of FT3 level (Table 2). The liver fat content (p for trend < 0.05) gradually decreased with the increasing of FT3. The liver fat content in the 3rd tertile (4.01 ± 2.43%) was significantly lower than that in 1st tertile (6.75 ± 3.45%) and 2nd tertile (6.14 ± 4.34%); The prevalence of NAFLD also showed a gradual downward trend (p for trend < 0.05). The prevalence of NAFLD in the 3rd tertile (1/38) was significantly lower than that in the 1st tertile (10/29), but there was no statistical difference between the 3rd tertile and 2nd tertile (3/36). (Fig. 1 a-b). In addition, the levels of BMI, TG, TC, and LDL-c gradually decreased, and the levels of ALT and AST gradually increased with the increasing of FT3 (p for trend< 0.01). There were no significant differences in gender, Wc, blood pressure level, FBG, fasting insulin, UA, IL-6, HCRP and HOMA-IR among the three groups (*p* > 0.05). After adjustment for age, gender, and BMI, the association of liver fat content still reached the statistical significance (*p* < 0.01).

**Table 2:** Clinical characteristics of all cases by tertiles of free triiodothyronine

FT3	T1(≦12.70 pmol/L)	T2(12.71 ~ 25.80 pmol/L)	T3(>25.80 pmol/L)	P for trend
Age (years)	49.9 ± 11.9	46.1 ± 13.8	40.1 ± 10.8※☆	0.002
Gender(M/F)	14/25	11/28	9/30	0.455
FT4(pmol/L)	25.28 ± 10.26	48.41 ± 14.11※	88.78 ± 17.28※☆	< 0.001
TSH (uIU/ml)	0.01 (0.005,1.470)	0.005 (0.005, 0.010)※	0.005 (0.005, 0.010)※	< 0.001
BMI (kg/m^2^)	23.41 ± 3.30	23.40 ± 3.41	20.85 ± 2.82※☆	< 0.001
Waist circumference (cm)	83.17 ± 8.82	81.43 ± 10.19	77.79 ± 8.95※	0.113
SBP (mmHg)	121.46 ± 13.26	124.72 ± 10.94	121.38 ± 16.68	0.480
DBP (mmHg)	74.92 ± 9.10	75.79 ± 8.54	72.26 ± 10.21	0.221
FBG (mmol/L)	5.01 ± 1.30	5.01 ± 0.79	5.25 ± 1.09	0.541
f-INS (mU/L)	8.30 (5.50, 9.70)	7.90 (5.80, 11.1)	9.20 (7.00, 11.00)	0.367
TG (mmol/L)	1.03 ± 0.48	1.14 ± 0.58	0.79 ± 0.28※☆	0.006
TC (mmol/L)	3.91 ± 0.85	3.47 ± 0.77※	2.94 ± 0.43※☆	< 0.001
LDL (mmol/L)	2.23 ± 0.63	1.76 ± 0.62※	1.36 ± 0.38※☆	< 0.001
IL6(pg/ml)	2.20 (2.00,3.00)	2.10 (2.00,2.58)	2.60 (2.10,3.38)	0.352
HCRP (mg/L)	0.80 (0.40,1.98)	0.70 (0.30,2.20)	1.00 (0.45,2.15)	0.389
UA (umol/L)	295.90 ± 77.90	282.28 ± 56.85	300.68 ± 72.63	0.486
ALT(U/L)	24.92 ± 19.76	36.79 ± 26.95※	48.69 ± 43.93※	0.006
AST(U/L)	21.46 ± 10.29	26.36 ± 13.41	33.62 ± 21.91※	0.004
Liver fat content(%)	6.75 ± 3.45	6.14 ± 4.34	4.01 ± 2.43※☆	0.002
With NAFLD/without NAFLD	10/29	3/36	1/38※	0.004
HOMA-IR	1.70 (1.11, 2.64)	1.76 (1.19, 2.55)	2.04 (1.63, 2.54)	0.289

※*P* <0.05 versus T1 group,; ☆ *P* <0.05 versus T2 group

Significant correlation was found between FT3 and liver fat content (R = -0.328, *P* < 0.01), the correlation was significant after adjusting for age, gender and BMI (R = -0.245, *P* < 0.01) (Fig. 1c, Table 3). Pearson correlation analysis showed that FT3 was negatively correlated with BMI, Wc, TG, TC and LDL, and positively correlated with ALT and AST (Table 3). The association of FT3 with TC, LDL, ALT and AST were still significant after adjusting for age, gender and BMI (*P* < 0.01).

**Table 3:** Pearson correlation analysis of factors associated with FT3 and liver fat content

index	Pearson correlation analysis (*P*-value)				Adjusted for age, gender (*P*-value)				Adjusted for age, gender, BMI (*P*-value)			
	Liver fat content		FT3		Liver fat content		FT3		Liver fat content		FT3	
	R	*P*-value	R	*P*-value	R	*P*-value	R	*P*-value	R	*P*-value	R	*P*-value
Age (years)	NS		−0.315	0.001	–	–	–	–	–	–	–	–
BMI (kg/m^2^)	0.337	< 0.001	− 0.347	< 0.001	0.321	< 0.001	−0.292	0.002	–	–	–	–
Waist circumference (cm)	0.300	0.006	−0.219	0.049	0.293	0.009	NS		NS		NS	
SBP (mmHg)	NS		NS		NS		NS		NS		NS	
DBP (mmHg)	NS		NS		NS		NS		NS		NS	
FT3(pmol/L)	−0.328	< 0.001	–	–	−0.316	0.001	–	–	−0.245	0.009	–	–
FT4(pmol/L)	−0.305	0.001	0.937	< 0.001	−0.291	0.002	0.930	< 0.001	−0.216	0.021	0.923	< 0.001
TSH (uIU/ml)	NS		−0.341	< 0.001	NS		−0.325	< 0.001	NS		−0.275	0.003
TG (mmol/L)	0.284	0.002	−0.239	0.011	0.274	0.004	−0.191	0.046	0.209	0.029	NS	
TC (mmol/L)	0.331	< 0.001	−0.547	< 0.001	0.323	0.001	−0.519	< 0.001	0.286	0.003	−0.495	< 0.001
LDL (mmol/L)	0.346	< 0.001	−0.543	< 0.001	0.333	< 0.001	−0.513	< 0.001	0.301	0.001	−0.492	< 0.001
IL6(pg/ml)	NS		NS		−0.194	0.048	NS		NS		NS	
HCRP (mg/L)	NS		NS		−0.240	0.012	NS		−0.215	0.026	NS	
UA (umol/L)	NS		NS	NS	NS		NS	NS				
ALT(U/L)	NS		0.310	0.001	NS		0.338	< 0.001	NS		0.336	< 0.001
AST(U/L)	NS		0.342	< 0.001	NS		0.374	< 0.001	NS		0.375	< 0.001
HOMA	NS		NS		NS		NS		NS		NS	

As shown in Table 4, multiple linear regression analysis was used to analyze the independent risk factors associated with liver fat content in hyperthyroidism patients. Model 1 included FT3, FT4, TSH, and BMI, and adjusted for age and gender. The results showed that FT3 (*p* < 0.01) and BMI (*p* < 0.01) were independently correlated with the liver fat content. The full model 2 further included the FT3, FT4, TSH, TG, CHOL, LDL, systolic blood pressure, diastolic blood pressure, UA, IL - 6, HCRP as the independent variables, and adjusted for age, gender and BMI. The results showed that FT3 (*p* < 0.05) and TG (*p* < 0.05) were independently correlated with liver fat content.

**Table 4:** Multiple linear regression analysis of independent factors associated with liver fat content

Dependent variable	Model 1^a^			Model 2^b^		
	Independent variable	β	*P*-value	Independent variable	β	*P*-value
Liver fat content	FT3	−0.072	0.009	FT3	−0.059	0.016
	BMI	0.281	0.007	TG	1.461	0.013

^a^Model 1 FT3, FT4, TSH, and BMI were included as the independent variables, adjusted with age and gender; ^b^ Model 2 FT3, FT4, TSH, TG, CHOL, LDL, systolic blood pressure, diastolic blood pressure, UA, IL-6, and HCRP were included as the independent variables, adjusted with age, gender and BMI

In the binary logistic regression analysis (Table 5), after adjustment with age and gender, FT3, FT4, TSH, and BMI tertiles were used and independent variables, and the results showed FT3 (OR 0.297, 95% CI.0.106 ~ 0.832), and BMI (OR 4.585, 95% CI.1.488 ~ 14.128) was independently associated with NAFLD. In full Model 2, FT4, TSH, TG, CHOL, LDL, Systolic blood pressure, diastolic blood pressure, UA, IL6, and HCRP tertiles were included, adjusted variable of age, gender and BMI. The results showed FT3 (OR 0.267, 95% CI.0.087 ~ 0.817) were still independently associated with NAFLD.

**Table 5:** Binary logistic regression analysis of risk factors for NAFLD

Binary logistic regression analysis	Independent variable	*P*-value	OR	95% C.l.
Model1^a^	FT3	0.021	0.297	0.106–0.832
	BMI	0.008	4.585	1.488–14.128
Model2^b^	FT3	0.021	0.267	0.087–0.817

^a^ Model 1 tertiles of FT3, FT4, TSH, and BMI were included as the independent variables, adjusted with age and gender; ^b^ Model 2 tertiles of FT3, FT4, TSH, TG, CHOL, LDL, systolic blood pressure, diastolic blood pressure, UA, IL-6, and HCRP were included as the independent variables, adjusted with age, gender and BMI

## Discussion

The present study reported that the prevalence of NAFLD was 11.97% in 117 clinical hyperthyroidism patients. Liver fat content was closely related to the FT3 levels in this population, and this association was independent of metabolic components and inflammatory factors.

The prevalence of NAFLD is 27.4–33.1% in euthyroidism population, and 35.7–36.3% in hypothyroidism population [1–3]. However, there are extremely limited reports on the prevalence of NAFLD in hyperthyroid population. From Rotterdam Study, the prevalence of NAFLD was 21.5% in hyperthyroidism subjects, and a decreasing trend of NAFLD prevalence was found in different thyroid status from hypothyroidism, euthyroidism to hyperthyroidism [20]. NAFLD risk decreased gradually from hypothyroidism to hyperthyroidism. However, 114 subjects were in subclinical status, there were only 7 clinical hyperthyroidism cases were included in Rotterdam Study. Our data showed that the prevalence of NAFLD 11.95% in clinical hyperthyroidism subjects, which was lower than subclinical hyperthyroidism and other thyroid status population. To be consistent of our findings, a case also reported that hyperthyroidism improved the pathological condition of NASH [21].

The results of the present study indicated a negative linear association between FT3 levels and NAFLD in this specific hyperthyroidism population. Several study demonstrated the association of thyroid hormone and NAFLD in different thyroid status population. The serum thyroxin (TT4) concentration in subjects with hepatic steatosis was reduced in subclinical and clinical hypothyroidism subjects [10]. Subclinical hypothyroidism and low-normal thyroid function are associated with NASH and fibrosis according to the TSH levels [22].From lifeline cohort study, higher FT3is associated with NAFLD in euthyroid subjects [23].Higher FT4 levels were associated with a decreased risk of NAFLD, and higher thyroid-stimulating hormone levels were associated with increased risk of having clinically relevant fibrosis in NAFLD in Rotterdam study [20]. Our study demonstrated that FT3 level was independently associated with decreased liver fat content in the hyperthyroidism population. TSH levels were significantly suppressed in hyperthyroidism subjects, which can not accurately reflect the thyroid function. In addition, FT3 activity is much higher than FT4 [24, 25], so it is more authentic to regard FT3 level as an indicator of thyroid function in patients with hyperthyroidism. Therefore, in hyper-metabolic hyperthyroid population, FT3 is independently associated with liver fat content, while in hypothyroid or euthyroid population, TSH or FT4 and NAFLD are closely related.

The mechanisms about the effect of thyroid hormone levels on liver fat content and NAFLD remain unclear. Study of rodent models have demonstrated that thyroid receptor β agonist MB07811 can reduce hepatic steatosis [12]. Thyroid hormone induces intrahepatic lipolysis via activation of autophagy [26]. Previous studies on the hypothyroidism patients indicated that lower thyroid hormone caused insulin resistance, metabolic disorders, and NAFLD [27, 28]. Our results indicated that thyroid hormone further reduced liver fat content in the condition of hyperthyroidism. Thyroid hormone may promote body fat consumption, and reduce body weight, it may also directly impact on the liver, accelerating intrahepatic fat clearance, this process was independent from metabolic factors and inflammatory factors.

Previous studies have shown hypothyroidism is related to NAFLD, and the level of thyroid hormone in hypothyroidism or euthyroidism population is closely related to liver fat content. Our study also showed that the prevalence of NAFLD in hyperthyroid populations is 11.97%, which was lower than the 27.4–33.1% in euthyroidism population as indicated in previous studies. Our study further expanded the study population and explored the effect of pathologically elevated thyroid hormones on NAFLD in patients with hyperthyroidism. The results showed that FT3 in patients with hyperthyroidism was significantly negatively correlated with liver fat content and was independent from other well-established NAFLD-related risks factors including BMI, TG, CRP, IL-6, etc. These findings expanded the significant association of thyroid hormone and NAFLD in hypothyroidism and euthyroidism population, elevated thyroid hormone levels can reduce liver fat content regardless of the thyroid functional status of the population.

There are still some limitations in this study: 1) Lack of follow-up data in this cross-sectional study; 2) The sample size was not estimated before the study, and the sample size was small. Due to the characteristics of the disease, the gender distribution of the included cases was unequal; 3) The study is limited to clinic-based setting which may be potentially responsible for bias in the results obtained; 4) Ler biopsy or liver magnetic resonance spectroscopy (MRS) were not used to accurately detect liver fat content. The results above still need to be further confirmed by improving the experimental design and following up closely.

## Conclusion

In conclusion, The present study reported that the prevalence of NAFLD was 11.97% in 117 clinical hyperthyroidism patients. In addition, the prevalence and liver fat content significantly decrease with the elevation of FT3 levels in this population. These results may provide new evidence in the role of thyroid hormone on the regulation of liver fat content and NAFLD.

## Data Availability

The data that support the findings of this study are available from the corresponding author upon reasonable request.

## Abbreviations

NAFLD: Non-alcoholic fatty liver disease
FT3: Free triiodothyronine
FT4: Free thyroxine
NASFL: Non-alcoholic simple fatty liver
NASH: Nonalcoholic steatohepatitis
FFA: Free fatty acids
TSH: Thyroid stimulating hormone
Wc: Waist circumference
BMI: Body mass index
FBG: Fasting blood glucose
TC: Total cholesterol
TG: Triglyceride
HDL-C: High-density lipoprotein cholesterol
LDL-C: Low-density lipoprotein cholesterol
ALT: Alanine aminotransferase
AST: Aspartate aminotransferase
UA: Uric acid
TRAb: Thyrotrophin receptor antibody
f-INS: Fasting plasma insulin
HCRP: Hyper-sensitive C-reaction protein
HOMA-IR: Insulin resistance index
ROI: Region of interest
